# Effects of Corn and Broken Rice Extrusion on the Feed Intake, Nutrient Digestibility, and Gut Microbiota of Weaned Piglets

**DOI:** 10.3390/ani12070818

**Published:** 2022-03-23

**Authors:** Yong Zhuo, Yingyan Huang, Jiaqi He, Lun Hua, Shengyu Xu, Jian Li, Lianqiang Che, Yan Lin, Bin Feng, De Wu

**Affiliations:** Animal Nutrition Institute, Sichuan Agricultural University, Chengdu 611130, China; zhuoyong@sicau.edu.cn (Y.Z.); abby_hyy@163.com (Y.H.); hejiaqi96@hotmail.com (J.H.); hualun0516@163.com (L.H.); shengyu_x@hotmail.com (S.X.); lijian522@hotmail.com (J.L.); clianqiang@hotmail.com (L.C.); linyan936@163.com (Y.L.); fengbin@sicau.edu.cn (B.F.)

**Keywords:** extruded broken rice, extruded corn, weaned piglets, feed intake, microbiota

## Abstract

**Simple Summary:**

Extruded cereals are largely used in newly weaned piglet diets to increase nutrient digestibility and palatability. Our findings showed that corn and broken rice extrusion diets generated negative effects on average daily feed intake (−63.5 g/day, *p* = 0.054) and average daily gain (−60.6 g/d, *p* = 0.015) in weaned piglets. Decreased feed intake was associated with increased plasma levels of the gut-derived hormones, glucagon-like peptide-1 (GLP-1) and peptide YY (PYY), which may have been attributed to increased microbiota pathogen abundance, including *Sarcina*, *Clostridium_sensu_strictio_1*, and *Terrisporobacter*, and decreased short-chain fatty acid-producing microbiota, such as *Lactobaillaceae* and *Bifidobateriaceae*. Our results showed that extruded cereals should be used cautiously when formulating diets for newly weaned piglets.

**Abstract:**

In this study, we investigated the effects of corn and rice extrusion diets on feed intake, nutrient digestibility, and gut microbiota in weaned piglets. Animals were divided into four dietary groups and fed a controlled diet containing (1) 62.17% corn (CORN), 15% soybean, 10% extruded full-fat soybean, and 6% fishmeal (2) half the corn replaced by extruded corn (ECORN), (3) broken rice (RICE), and (4) extruded broken rice (ERICE) for 28 days. Rice supplementation increased dry matter total tract digestibility and gross energy. Extruded cereals generated a lower average daily feed intake (ADFI) at 15–28 and 1–28 days, decreased average daily growth (ADG) at 15–28 and 1–28 days, and a lowered body weight (BW) on day 28, regardless of cereal type. Dietary extruded cereals increased the appetite-regulating hormones glucagon-like peptide-1 (GLP-1) and peptide YY (PYY). Piglets fed extruded cereals displayed low short-chain fatty acid (SCFA) levels in plasma and low *Lactobaillaceae* and *Bifidobateriaceae* levels in feces, whereas a higher abundance of the potential pathogens *Sarcina*, *Clostridium_sensu_strictio_1* and *Terrisporobacter* was observed. Piglets fed extruded cereals displayed significantly lower gas and SCFA levels during in vitro fermentation. Combined, 50% corn substituted with extruded corn or broken rice decreased piglet growth performance, possibly by altering their microbiota.

## 1. Introduction

Decreased feed intake and growth performance are common in weaning piglets, and may be attributed to numerous stresses including mixing and moving, dam separation, and changes in diet and feeding systems. Thus, new nutritional strategies aimed at stimulating feed intake and improving digestive functions during this period are required in swine production [1]. Rice is one of the most abundant cereals, however, as it is a staple food for humans, its use as an animal feed ingredient has been restricted [2]. Broken rice, a by-product of rice processing, has received considerable attention since its inclusion in piglet diets improved growth performance [3,4,5] and reduced the mortality of nursery piglets [6]. Broken rice exhibits greater nutrient digestibility compared with corn [7], thus, it has a higher postprandial glycemic curve [8]. High glycemic index (GI) carbohydrates promote higher glucose and insulin secretion peaks when compared with low GI carbohydrates, which ultimately results in differences in feed intake [9]. 

Extruded cereal-based products, such as extruded corn, broken rice, and wheat, are widely used in swine production, as they improve nutrient digestibility and feed intake following weaning [6,10]. In addition, the extrusion process induces several chemical and structural changes in cereal nutrients (e.g., starch), leading to changes in digesta retention times in the intestine [11]. Extruded cereal grains may also contain resistant starch that is not easily digested or absorbed in the small intestine, but rather passes onto the large intestine. Thus, cereal grain extrusion diets enhance microbiota access to undigested carbohydrates and microbial fermentation in the hindgut, which putatively affects feed intake via specific appetite regulatory factors, such as short-chain fatty acids (SCFAs), glucagon-like peptide-1 (GLP-1), and peptide YY (PYY) [12]. However, it is unclear how cereal extrusion diets interact with the gut microbiota to influence host feeding physiology.

Therefore, this study was conducted to investigate the effects of different grains (corn or broken rice) and the extrusion processing method (extruded corn or extruded broken rice) on feed intake, nutrient digestibility, and the microbiota in weaned piglets.

## 2. Materials and Methods

### 2.1. Ethical Considerations

This study was performed at Sichuan Agricultural University. The experimental protocol was in accordance with the animal care and use committee guidelines of our University (Ethics Approval Code: 20184006), and followed national laws and National Research Council (NRC) guidelines for the care and use of laboratory animals.

### 2.2. Preparation of Extruded Cereals

Cereal extrusions were prepared as previously described [10]. Briefly, broken early Indica rice from the Chongqing province (China) and corn from Northeast China were ground using a hammer mill and a 2.5 mm screen, and extruded using a single-screw extruder (model TPH200C, Jiangsu Muyang Group, Yangzhou, China). Samples were steam-cooked at 80 °C for 20 s, then extruded at 125 °C and 100 °C for 5 s each. After this, corn and broken rice were cooled using a counter-flow cooling procedure and ground through a 2.5 mm screen to generate mashed cereal. Intact corn and broken rice were used as controls. 

### 2.3. Animals, Diets, and Management

Sixty-four crossed Duroc × (Landrace × Yorkshire) piglets, weaned at day 28 (±1 day) and having a body weight (BW) of 7.11 kg (±0.16 kg) were used in a 28-day feeding study. Piglets were randomly allocated to one of four dietary treatments, with eight replicates (pens) per treatment group. There were two piglets in each pen (one barrow and one gilt). The four study diets included one control diet containing 62.17% corn (CORN), and three diets with half the corn replaced by: extruded corn (ECORN); broken rice (RICE); and extruded broken rice (ERICE) (Table 1). Piglets were adapted to a common prestarter diet for 3 days. Each pen was equipped with one self-feeder and two nipples supplying water. Piglets were fed and watered ad libitum and provided with feed four times per day (8:00, 12:00, 16:00, and 20:00). Feeding conditions were observed for an average of 2 h to ensure surplus feed in feeding troughs was not less than 25% of the tank volume. The environmental temperature was maintained at 24–26 °C. Diets were supplied in mashed form and formulated to contain equal quantities of digestible energy, crude protein, and lysine. Diets were formulated to meet or exceed the nutrient requirements as recommended by the NRC [13].

### 2.4. Sample Collection

Before study commencement, diets were sampled and stored at −20 °C for chemical analysis. Piglets were weighed on days 1, 14, and 28. Feed consumption was recorded daily on a pen basis to calculate the average daily feed intake (ADFI), the average daily growth (ADG), and the feed conversion ratio (F:G).

In the evening of days 13 and 27, the remaining feed in troughs was weighed and piglets fasted overnight. At 9:00 a.m. on days 14 and 28 of experiment, one piglet (barrow) in each pen was selected for blood collection (while in the fasting state) and marked on the back to denote selection. Blood samples from the jugular vein were collected into heparinized vacuum tubes. Piglets were provided with feed after blood collection, and additional blood samples were collected from marked piglets at 1 h, 2 h, and 4 h post-feeding. Blood samples were centrifuged at 3000× *g* for 15 min at 4 °C, with plasma stored at −20 °C. The plasma samples collected before feeding and 1 h and 2 h after feeding on day 28 were determined for concentrations of GLP-1 and PYY. The plasma samples collected before feeding and 4 h after feeding were determined for the level of acetate, propionate, and butyrate.

On days 13 and 27, one piglet per pen was selected to provide fecal samples. Feces were collected into sterile tubes, immediately frozen in liquid nitrogen, and stored at −80 °C for SCFA and bacterial analysis. Feces were collected under sterile conditions during sampling. 

From day 21, the indigestible marker, chromium (III) oxide (Cr_2_O_3_), was added to feed at 0.3% to measure apparent total tract digestibility (ATTD). After a 3-day adaption, freshly voided fecal samples from each pen were collected by hand into plastic bags on days 25–28, and 10% hydrochloric acid was added (10 mL/100 g feces) to fix excreta nitrogen. Samples were maintained on ice and stored at −20 °C. Then, samples were dried in a forced-air oven at 60 °C for 5 days. Dried samples were pooled for each pen and ground using a heavy-duty blender for dry matter (DM), crude protein (CP), ether extract (EE), gross energy (GE), crude ash (ASH), and crude fiber (CF) analysis.

The diarrhea index was measured by the appearance of feces monitored at 9:00, 14:00, and 20:00 daily. The fecal score was defined as 0 for forming or granular feces, 1 for soft but forming feces, 2 for thick, unseparated, unformed liquid feces, and 3 for unshaped, separated liquid feces. 

### 2.5. In Vitro Microbial Fermentation

Fecal microbiota were collected from each pen at day 28 and used in in vitro fermentation trials, conducted as previously described, but with minor modifications [14]. Briefly, feces were rectally collected, placed in plastic bags, anaerobically preserved in a foam box filled with ice, and transferred to the laboratory within 2 h. Feces (~70 g) were weighed and mixed with 0.9% sterile saline at 1:5 (g/mL). Samples were homogenized for 30 s using a hand mixer and filtered through four sterile cheesecloth layers to collect filtrates, which were transferred to air-tight plastic syringes and incubated in a water bath at 39 °C. The fermentation medium contained the following (per liter): peptone, 0.2 g; NH_4_HCO_3_, 0.4 g; NaHCO_3_, 35 g; Na_2_HPO_4_12H_2_O, 9.45 g; K_2_HPO_4_, 6.2 g; MgSO_4_7H_2_O, 0.6 g; CaCl_2_2H_2_O, 13.2 mg; MnCl_2_4H_2_O, 10 mg; CoCl_2_6H_2_O, 1 mg; FeCl_3_6H_2_O, 8 mg; L-Cysteine HCL, 1 g; and pectin, 120 mg. Syringes were sealed with glycerinum. Fermentation gases were recorded at 0, 3, 6, 9, 12, 18, 21, 24, 36, 48, 60, and 72 h using a glass syringe. Blanks containing inocula without medium were used to calculate background levels. In vitro fermentation characteristics, including the final asymptotic gas volume in feces (*V*_F_), initial fractional rate of degradation at t-value = 0 (*FRD*_0_), fractional rate of gas production at a particular time (*K*), and half-life to asymptote (*T*_1/2_), were determined as previously described [15]. The in vitro production of the SCFAs, acetate, propionate, butyrate, isobutyrate, valerate, and isovalerate were also determined.

### 2.6. Chemical Analysis and Intakes of Digestible Nutrients

The degree of starch gelatinization (%) as a proportion of total starch for the ingredients corn, extruded corn, broken rice, and extruded broken rice was determined by enzymatic hydrolysis as described previously [16]. This method involved the incubation of amyloglucosidase (A7095; Sigma-Aldrich, St Louis, MO, USA) with 0.2 g sample followed by photometrical product measurement of the reaction between glucose and the copper reagent, ferricyanide. The reaction followed the principle that gelatinized starch was digested more easily by amyloglucosidase to form glucose. Apparent total tract digestibility (ATTD) was determined using Cr_2_O_3_ as an indigestible marker. ATTD was evaluated in each cage individually. All diet and feces samples were analyzed in duplicate for Cr (method 990.08), DM (method 930.15), ASH (method 942.05), EE (method 945.16), CP (method 990.03), and CF (method 920.98) according to AOAC procedures (1995). The GE of feed and fecal samples was determined using an adiabatic oxygen bomb calorimetry apparatus (Parr Instrument Co., Moline, IL, USA). ATTD was calculated using the following formula: ATTD (%) = (100 − A1/A2 × F2/F1 × 100), where A1 = Cr content in feed; A2 = Cr content in feces; F1 = nutrient content in feed; and F2 = nutrient content in feces. The intakes of ATTD nutrients were calculated by the feed intake during days 22–28 of the experiment, and the ATTD of nutrients was determined during days 25–28 of the experiment.

### 2.7. Determining Appetite Hormone Plasma Levels

GLP-1 and PYY plasma levels from marked piglets recorded before feeding and 1 h and 2 h after feeding on day 28 were assessed using commercial kits (BIM Inc., San Francisco, CA, USA) according to the manufacturer’s instructions. Ghrelin and insulin plasma levels recorded before feeding and 4 h after feeding on day 28 were assessed using commercial kits (Jiangsu Meimian Industry Co., Ltd., Nanjing, China) according to the manufacturer’s instructions. Glucose levels before feeding and 1 h, 2 h, and 4 h post-feeding were assessed using commercial kits (Sichuan Maker Biotechnology Inc., Chengdu, China) on an automatic biochemical analyzer (Hitachi 7020, Hitachi High-Technologies Corporation, Tokyo, Japan). For glucose, the minimal detection limit was 0.02 mmol/L.

### 2.8. Determining SCFA Levels

The SCFA pretreatment of plasma, fecal samples, and fermentation media was conducted according to a previous method [17]. SCFA levels of acetate, propionate, and butyrate in plasma and feces were determined by gas chromatography (CP-3800GC, Varian, Inc., Walnut Creek, CA, USA), following instructions from a previous method [18].

### 2.9. Fecal Microbiota Analysis

Fecal samples collected on day 28 were analyzed for microbial diversity by extracting microbial DNA using QIAamp Fast DNA Stool mini kits (Qiagen, Hilden, Germany) according to the manufacturer’s instructions [19]. DNA concentrations and integrity were assessed on a Nanodrop-1000 and 0.8% agarose gel electrophoresis, respectively. The fusion primers, 338F 5′-ACTCCTACGGGAGGCAGCA-3′ and 806R 5′-GGACTACHVGGGTWTCTAAT-3′ with dual index, were used to amplify the V3–V4 region of bacterial 16S rRNA over 27 cycles of 95 °C for 30 s, 55 °C for 30 s, 72 °C for 45 s, and a final extension at 72 °C for 10 min. Amplicon sequencing was performed on an Illumina MiSeq platform (Illumina, San Diego, CA, USA). Paired-end reads from clean data sets were assembled into tags using FLASH (v.1.2.11) [20]. Microbial diversity was assessed using QIIME software and operational taxonomic units (OTUs) were defined at ≥97% sequence homology. Taxonomic composition was generated using QIIME-UCLUST based on the Ribosomal Database Project. Principal coordinate analysis plots (PCoA) were generated at the genus level and were based on the Bray–Curtis dissimilarity. Microbial 16S rRNA sequencing analysis was conducted as previously described [21]. 

### 2.10. Statistical Analysis

Data were analyzed using a completely randomized study design with a 2 × 2 factorial treatment arrangement. Data normality and variance homogeneity were evaluated by Shapiro–Wilk and Levene tests, respectively. Data were analyzed by a mixed-procedure approach using SAS 9.4 (SAS Institute Inc., Cary, NC, USA). The fixed effects were cereal type, the extrusion process, and interactions between cereal sources and the extrusion process. The plasma concentrations of glucose, ghrelin, acetate, propionate, butyrate, total SCFAs, GLP-1, and PYY before feeding and at different intervals after feeding were analyzed using the MIXED procedure in SAS 9.4:

*Y*_ijkl_ = *µ* + *α*_i_ + *β*_j_ + *γk* + (*αβγ*)_ijk_ + *t*_Ɩ_ + *ε*_ijkl_

where *Y*_ijkl_ is the response variable, *µ* is the overall mean; and *α*_i_, *β*_j_, and *γk* are fixed effects of cereal type (corn or broken rice), extrusion process, and time of sample collection, respectively. (*αβγ*)_ijk_ is the interaction among fixed effects, *t*_Ɩ_ is the random effect of the gilt to account for repeated measurements within a piglet and *ε*_ijkl_ is the residual error. Results were expressed as the mean ± standard error. Differences were considered significant at *p* < 0.05, whereas 0.05 ≤ *p* < 0.10 values indicated a tendency.

## 3. Results

### 3.1. Gelatinization Properties of Extruded Corn and Broken Rice

The degrees of starch gelatinization of corn, extruded corn, broken rice, and extruded broken rice are shown (Table 2). Starch gelatinization levels in unprocessed and extruded corn were 12.74% and 75.48%, respectively. Starch gelatinization levels in unprocessed and extruded broken rice were 14.76% and 93.46%, respectively.

### 3.2. Piglet Growth Performance

The growth performance of weaned piglets is shown (Table 3). The final BW of piglets on days 14 and 28 was not affected by cereal type (*p* > 0.05) or interactions between cereal type and the extrusion process (*p* > 0.05), but BW showed a decreasing tendency due to the extrusion process on day 28 (16.5 vs. 15.1 kg, *p* = 0.081). The ADFI was decreased by the extrusion process at days 15–28 (816.1 vs. 717.9 g/d, *p* = 0.013) and days 1–28 (612.2 vs. 548.7 g/d, *p* = 0.054). The ADFI showed an elevated tendency due to broken rice inclusion at days 15–28 (734.1 vs. 799.9 g/d, *p* = 0.085). The ADG was decreased by the extrusion process at days 15–28 (439.3 vs. 368.3 g/d, *p* = 0.006) and days 1–28 (334.85 vs. 274.2 g/d, *p* = 0.015). F:G and diarrhea index values were unaffected by dietary treatments (*p* > 0.05). 

### 3.3. ATTD of Nutrients and Intake of Digestible Nutrients

The ATTD of nutrients is shown (Table 4). The ATTD of DM in piglets was increased by broken rice (*p* = 0.021). The ATTD of CP was increased by the extrusion process (*p* = 0.042) and affected by interactions between cereal type and the extrusion process (*p* = 0.049), whereas the ATTD of CP in the CORN group was lower than the other groups (*p* < 0.05). The ATTD of EE showed a decreased tendency with the extrusion process (*p* = 0.055). The ATTD of GE showed an elevated tendency with broken rice inclusion (*p* = 0.086). The intakes of digestible DM, CP, and energy were significantly lower by extrusion process (*p* < 0.001), and the type of cereal grains affected the intakes of digestible CP (*p* = 0.09) and energy (*p* = 0.045) at days 22–28 of the experiment.

### 3.4. Plasma Concentrations of Blood Parameters

The glucose, insulin, and ghrelin concentrations in plasma collected at different times in piglets fed with different diets are presented in Table 5. The glucose concentrations in plasma were affected by the extrusion process (*p* = 0.035) and time of collection (*p* = 0.003), but not affected by cereal type, interaction of cereal type and extrusion, interaction of cereal type and time, or interaction of extrusion and time (*p* > 0.05). The insulin concentrations in plasma were affected by cereal type (*p* = 0.032). The ghrelin concentrations in plasma were affected by time of collection (*p* = 0.015) and the interaction of cereal type (*p* = 0.015) and time of collection (*p* = 0.018). 

**Figure 1:**
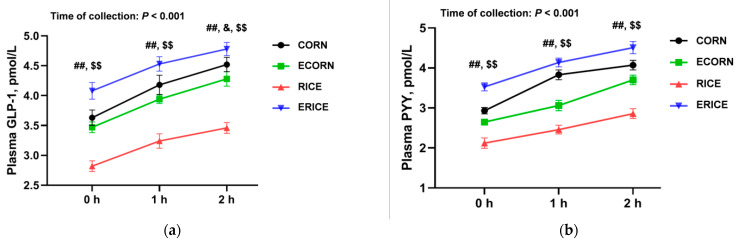
Effect of cereal types and extrusion processing on the plasma concentration of GLP-1 (**a**) and PYY (**b**) of weaned piglets. Values are means ± SEM, n = 8. $$, *p*_extrusion process_ *<* 0.01; ##, *p*_interaction_ *<* 0.01. &, *p*_cereal types_ *<* 0.05.

As shown in Figure 1a,b, both GLP-1 and PYY concentrations in plasma were affected by the extrusion process (*p* < 0.001), time of collection (*p* < 0.001), and the interaction of cereal type and extrusion process (*p* < 0.001), but not affected by cereal type, interaction of cereal type and time, interaction of extrusion and time, or the interaction of cereal type, extrusion, and time (*p* > 0.05). As shown in Figure 1a, GLP-1 plasma levels at 0 h (before feeding) and 1 h and 2 h after feeding were affected by the extrusion process and interactions between cereal type and the extrusion process (*p* < 0.01). GLP-1 plasma levels were similar between CORN and ECORN piglets (*p* > 0.05), but were greater in ERICE piglets when compared with RICE piglets (*p* < 0.05) at 0 h, 1 h, and 2 h after feeding. Cereal type also affected GLP-1 plasma levels at 2 h after feeding (*p* < 0.05). As shown in Figure 1b, PYY plasma levels at 0 h, 1 h, and 2 h after feeding were affected by the extrusion process and interactions between cereal type and the extrusion process (*p* < 0.01). Plasma PYY levels in CORN piglets were greater than ECORN piglets (*p* < 0.05), but in RICE piglets, levels were lower than ERICE piglets at 0 h, 1 h, and 2 h after feeding (*p* < 0.05). 

### 3.5. SCFA Levels in Plasma and Fecal Samples

As shown in Table 6, fecal acetate, propionate, butyrate, and the sum of these levels were similar between groups. The effects of cereal type and the extrusion process on SCFA plasma levels are shown (Table 7). The plasma concentrations of acetate and butyrate were decreased by RICE (*p* = 0.002 and *p* < 0.001, respectively) and time of collection (*p* = 0.030 and *p* = 0.001, respectively). The plasma concentrations of propionate were affected by the interaction of cereal type and time of collection (*p* = 0.038). Total SCFAs were affected by cereal types (*p* = 0.008).

### 3.6. In Vitro Fermentation Characteristics

**Figure 2:**
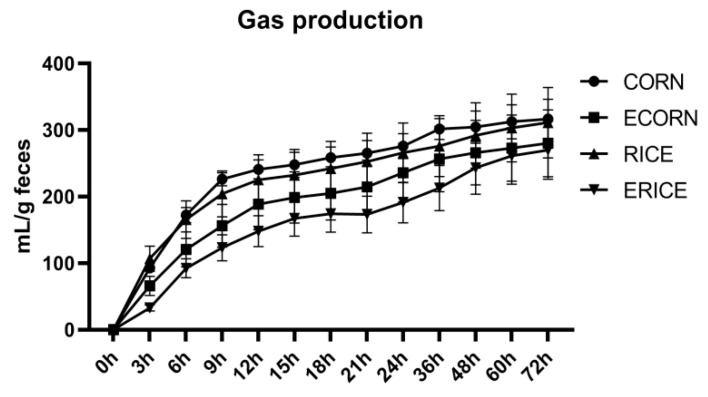
Effect of extrusion of corn and broken rice on gas production curve by fecal microbial fermentation. Treatments were corn as the main cereal type (CORN), or half of the corn replaced by extruded corn (ECORN), broken rice (RICE), and extruded broken rice (ERICE), respectively.

As shown in Figure 2, gas production dynamics differed significantly by dietary treatment. The *V*_F_, *K*, *FRD*_0_, and *T*_1/2_ parameters were used to indicate the in vitro fermentation characteristics (Table 8). *V*_F_ was significantly decreased by broken rice inclusion (*p* < 0.001) and affected by interactions between cereal type and the extrusion process (*p* = 0.001). *K*, *FRD*_0_, and *T*_1/2_ parameters were decreased by broken rice inclusion (*p* < 0.05) and the extrusion process (*p* < 0.001). SCFA levels at the end of in vitro fermentation (in culture medium) are shown (Table 8). Broken rice inclusion significantly decreased butyrate (*p* = 0.031) and valerate (*p* < 0.001) production. Extruded cereals lowered the production of acetate, butyrate, isobutyrate, valerate, isovalerate, and total SCFAs (*p* < 0.01). Propionate, butyrate, isobutyrate, valerate, and isovalerate production levels were affected by interactions between cereal type and the extrusion process (*p* < 0.01).

### 3.7. Fecal Microbial Diversity and Composition

#### 3.7.1. Sequence Analysis

A total of 3,720,720 raw reads were generated from 32 samples, with an average of 58,136 ± 6200 sequences per fecal sample. After removing low-quality sequences, 1,860,360 valid sequences with an average length of 414 base pairs (bp) were obtained. In total, 977 OTUs were generated, which identified 13 bacterial phyla and 202 bacterial genera.

#### 3.7.2. Alpha and β-Diversity 

**Figure 3:**
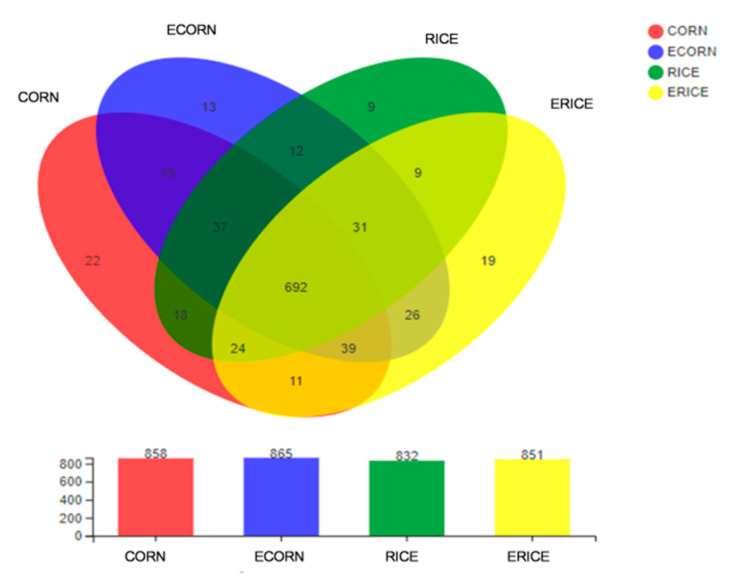
Venn diagram shows the unique and shared operational taxonomic units (OTUs) in different groups at the genus level. Treatments were corn as the main cereal type (CORN), or half of the corn replaced by extruded corn (ECORN), broken rice (RICE), and extruded broken rice (ERICE), respectively.

**Figure 4:**
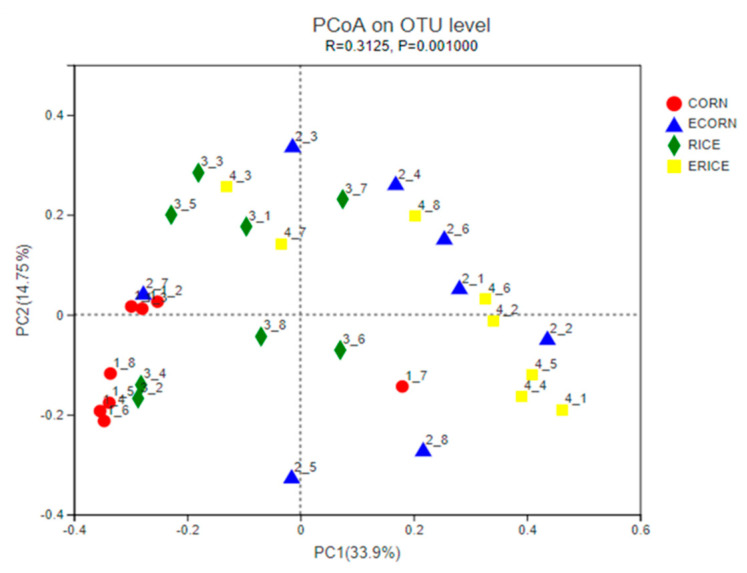
Principal coordinate analysis (PCoA) of bacterial community structures among the four groups. Treatments were corn as the main cereal type (CORN), or half of the corn replaced by extruded corn (ECORN), broken rice (RICE), and extruded broken rice (ERICE), respectively.

Alpha diversity, represented by Shannon’s diversity index, Simpson index, Chao1, Ace, and Coverage, was used to assess microbial diversity (Table 9). The extrusion process appeared to have higher levels of Chao1 and ACE index (0.05 ≤ *p* < 0.1). A Venn diagram identified shared and unique OTUs between groups (Figure 3). There were 858, 865, 831, and 851 OTUs identified in CORN, ECORN, RICE, and ERICE groups, respectively. The CORN group had 22 unique OTUs, the ECORN group had 13, the RICE group had 9, and the ERICE group had 19. PCoA (Figure 4) indicated an obvious separation between non-extruded groups (CORN and RICE groups) and extruded groups (ECORN and ERICE groups). 

**Figure 5:**
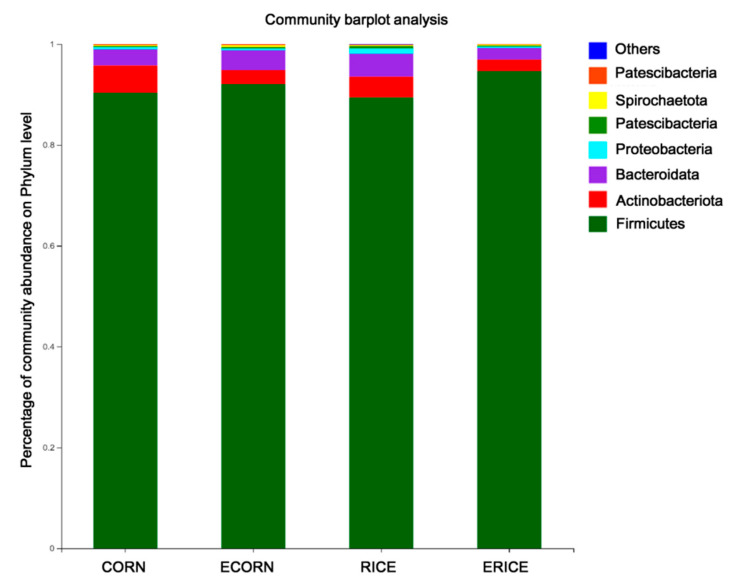
Analysis for differential bacteria at the Phylum level among four dietary treatments. Treatments were corn as the main cereal type (CORN), or half of the corn replaced by extruded corn (ECORN), broken rice (RICE), and extruded broken rice (ERICE), respectively. *n* = 8.

Taxonomic classifications of bacterial 16S rRNA at phylum levels on day 28 are shown (Figure 5). In total, 13 phyla and 83 families were identified in fecal samples on day 28. The most dominant bacterial phyla were *Firmicutes*, which accounted for 91% of total bacteria. Additionally, both CORN and RICE groups showed significantly higher *Actinobacteria* levels than ECORN and ERICE (*p* < 0.05). 

**Figure 6:**
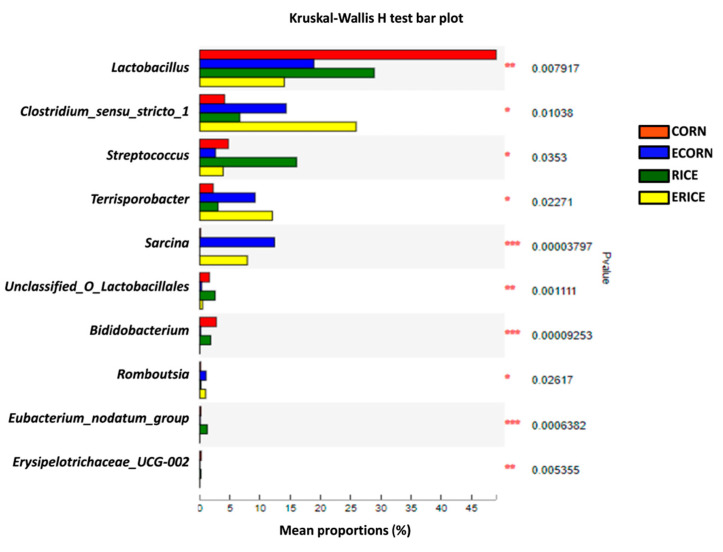
Analysis for differential bacteria at the family level among 4 dietary treatments. Treatments were corn as the main cereal type (CORN), or half of the corn replaced by extruded corn (ECORN), broken rice (RICE), and extruded broken rice (ERICE), respectively. *n* = 8.

The genera displaying significant effects by cereal type or extrusion process are shown (Figure 6). Kruskal–Wallis analyses were used to compare microbial composition at the genera level in the four groups, which revealed that *Lactobacillus*, *Clostridium_sensu_stricto_1*, *Streptococcus*, *Terrisporobacter*, *Sarcina*, *Unclassified_O_Lactobacillales*, *Bididobacterium*, *Romboutsia*, *Eubacterium_nodatum_group*, and *Erysipelotrichaceae_UCG-002* were differentially affected by dietary treatments (*p* < 0.05 or *p* < 0.01).

**Figure 7:**
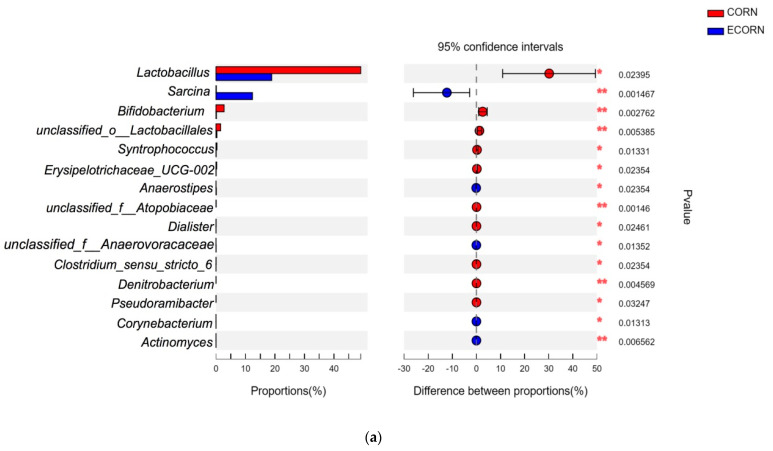

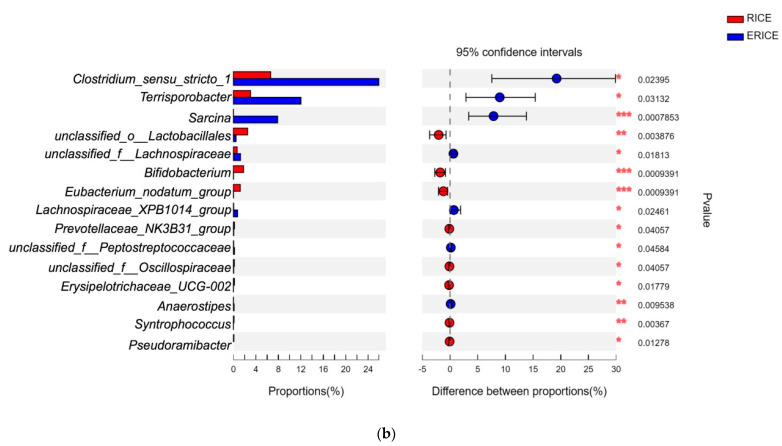
Differential abundance of bacteria between CORN and ECORN (**a**) and between RICE and ERICE (**b**). Treatments were corn as the main cereal type (CORN), or half of the corn replaced by extruded corn (ECORN), broken rice (RICE), and extruded broken rice (ERICE), respectively. * *p* < 0.05; ** *p* < 0.01; *** *p* < 0.001. *n* = 8.

To understand the effects of the extrusion process on fecal microbiota composition, the relative abundance of microbiota between CORN and ECORN groups and between RICE and ERICE groups were further investigated (Figure 7a,b). The relative abundance of *Lactobacillus*, *Bifidobacterium*, *unclassified_o_Lactobacillales*, *Syntrophococcus*, *Erysipelotrichaceae_UCG-002*, *unclassified_f_Atopobiaceae*, *Dialister*, *Clstridium_sensu_stricto_6*, *Denitrobacterium*, and *Pseudoramibacter* was significantly increased (*p* < 0.05), whereas *Sarcina*, *Anaerostipes*, *Dialister*, *Corynebacterium*, and *Actinomyces* abundance was significantly decreased in ECORN when compared with CORN piglets (*p* < 0.05, Figure 7a). *Clostridium_sensu_strictio_1*, *Terrisporobacter*, *Sarcina*, *unclassified_f_Lachnospiraceae*, *spiraceae_XPB1014_group*, *unclassified_f_peptostreptococcaceae*, and *Anaerostipes* populations were significantly decreased in the RICE group when compared with the ERICE group (*p* < 0.05). However, the RICE group showed a greater relative abundance of *unclassified_o_Lctobacillales*, *Bifidobacterium*, *Eubacterium_nodatum_group*, *Prevotellaceae_NK3B31_group*, *unclassified_f_Oscillospiraceae*, *Eryipelotrichaceae_UCG-002*, *Syntrophococcus*, and *Pseudoramibacter* when compared with the ERICE group (*p* < 0.05).

## 4. Discussion

In this study, our primary aim was to determine the influence of different grains (corn and broken rice) and an extruded processing diet (extruded corn/extruded rice) on feed intake, growth performance, and associated microbiota changes in weaned piglets. Broken rice is a favorable dietary ingredient due to its high starch and low fiber levels. It was observed that the ATTD levels of DM and GE in RICE and ERICE groups were greater than those in CORN and ECORN groups, respectively, in agreement with a previous study [10]. Increased ATTD values in the broken rice group may have been due to the lower size of rice starch relative to corn starch [22]. Rice starch has a smaller starch granule, lower non-starch polysaccharide levels, and lower amylose-to-amylopectin ratios than corn starch, thereby contributing to increased starch digestibility [23]. Since piglets in the RICE and ERICE groups had greater ATTD levels of nutrients as well as greater ADFI, their intakes of digestible nutrients were also greater, which might be the reason why they had greater performance that CORN and ECORN piglets. 

Previous studies reported that corn-to-rice substitution in piglet diets improved feed intake, ADG, and nutrient digestibility [2,24]. In the current study, the RICE group displayed the best growth performance, but no significant differences were observed when compared with the CORN group. This may have been due to differences in feed textures. In previous studies, experimental reagents included rice or brown rice, which contained rice bran, and were classified as “functional foods”. A previous study reported that nursery diet supplementation with 10% rice bran increased growth performance and feed efficiency, similar to conventional diets containing growth-promoting antibiotics [25].

A previous study also demonstrated that gelatinized starch exhibited greater digestibility than raw starch [26]. However, in the present study, corn and broken rice extrusion diets did not increase the growth performance of piglets. This may have been due to gelatinization and supplementation levels. A brown rice extrusion diet did not affect piglet ADG; in fact, it decreased the G:F ratio, which was possibly due to reductions in essential amino acid levels [27]. As previously reported, rice starch had the highest expansion ratio when compared with bean starch and corn starch [28]. Similarly, a previous study showed that cereal heat-processing exerted no beneficial effects on broiler performance [29]. The effect of supplementation of extruded material might be dependent on the feeding duration. For example, the feed conversion ratio (FCR) on days 8 to 14 tended to be improved in groups supplemented with non-extruded cereals; this observation was supported by the fact that pigs fed a corn- and rice-rich diet displayed catch-up growth in the second week following weaning [10]. Moreover, the benefits of extruded materials might be dependent on the types of cereal grains. When the feed ingredients contain high levels of antinutritional factors such as soybean, an extrusion treatment would exert a greater benefit. Therefore, a better use of extruded materials for weaned piglet feed should be identified.

The decreased growth performance of piglets fed extruded ingredients could be attributed to lower feed intake, as FCRs were not influenced by dietary treatments. To gain insights on why the feed intake of piglets in ECORN and ERICE groups were decreased, appetite-related metabolites and hormones were investigated. Blood glucose is a primary signal influencing appetite [30], and was unaffected by dietary treatments. Insulin is an orexigenic hormone that increases hunger sensations and heightens sucrose palatability, regardless of glucose plasma levels [31]. Large insulin responses after a high-GI meal cause postprandial glucose depletion, which in turn increases hunger symptoms and promotes body fat accumulation and BW gain. Corn, raw rice grains, and rice could be considered as high-GI ingredients for pigs [32]. In this study, insulin plasma levels were decreased by broken rice inclusion at 4 h after feeding. However, feed intake was unaffected by broken rice inclusion; thus, alterations in feed intake appeared to be unaffected by insulin in the present study.

The gastric hormone ghrelin produces hunger and crave pangs [33,34]. In pigs, serum ghrelin levels can indicate chronic changes in energy status. In this study, ghrelin plasma levels were similar before feeding. At 4 h after feeding, levels were higher in diets supplemented with broken rice (or extruded broken rice). This was possibly due to lower SCFA levels in piglets fed broken rice, as ghrelin plasma levels are negatively regulated by plasma SCFAs [35,36]. Moreover, it was previously shown that additional protein supplementation decreased ghrelin plasma levels, suggesting levels may be related to protein digestibility [37]. 

GLP-1 and PYY are the main gut-derived anorexigenic hormones that regulate feeding behavior. GLP-1 and PYY plasma levels were significantly elevated by extruded ingredients, and potentially explained decreased feed intake and growth performance. Interestingly, GLP-1 and PYY levels were mainly affected by gut functions; SCFA-mediated glucose homeostasis and energy metabolism may be stimulated by enteroendocrine L-cells, which produce GLP-1 and PYY. Thus, increased microbiota-derived SCFA levels could in turn stimulate enteroendocrine cells to release GLP-1 [37] and PYY [38], while decreasing ghrelin secretion [39]. In the present study, the nutrient ATTD and feed intake were combined (Table 4), and we found that extrusion also results in greater nutrient intake such as crude protein; thus, the dietary-extrusion-induced changes of appetite-related hormones could be attributed to the alteration of nutrient intake. 

Lowered SCFA plasma levels in rice diets may have arisen due to lower indigestible carbohydrate levels, such as fiber and non-starch polysaccharides in corn, which may have escaped digestion in the small intestine and reached the colon to undergo microbial fermentation. Indigestible carbohydrates modulate gut microbiota, as evidenced by higher *Lactobacillus* abundance, which is one of the most common lactic acid-producing bacteria in humans and animals [40]. 

Corn or rice extrusion diets decrease SCFA plasma levels, possibly due to chemical and structural changes in extruded cereals [41]. Extrusion of diet elevates carbohydrate and other nutrient digestibility in the small intestine and reduces the supply of fermentable substrates to the large intestine, causing lower SCFA levels [42]. The SCFA was mainly produced by the gut microbiota, and the differences of serum levels of SCFA could be attributed to alternations of microbiota [43]. In the present study, compared with the piglets fed with extruded cereal, the piglets in unextruded diet groups showed a higher abundance of *Actinobacteria*, a predominant commensal bacterium in the gut [44]. In the present study, we further compared the microbial abundance at the family level between CORN and ECORN, as well as those between RICE and ERICE. One of the most important findings was that more probiotics such as *Lactobacillus* and *Bifidobacterium* had colonized the gut of piglets fed non-extruded diets compared with piglets fed the extruded diets. *Bifidobacteria* use complex carbohydrates, which are difficult to digest by the host [45]. *Lactobacillus* inhibits the growth of some intestinal pathogens such as *Escherichia coli* [46]. *Unclassified_o_lactobacillales* is also an SCFA producer during polysaccharide fermentation [47], and was elevated in non-extruded dietary groups when compared with extruded groups. This explained elevated plasma butyrate levels in the former group. Their proliferation might exert beneficial effects on the health of piglets, as SCFA could act as an energy source of intestinal epithelial cells. On the other hand, extruded ingredients stimulated the relative abundance of *Sarcina*, *Clostridium_sensu_strictio_1*, and *Terrisporobacter*, which exhibit pathogenic potential. *Sarcina* causes fatal bloat in animals [48], and is associated with acute abomasal bloating in young lambs and calves [49]. Therefore, dietary extrusion may stimulate the colonization of pathogenic microbes as well as decrease the beneficial commensal microbes, and this might be the reason why dietary extrusion causes negative effects on feed intake for piglets.

Given the importance of SCFA in mediating the microbial effects on the host physiology, and the fact that determined fecal SCFA concentrations represented less than 3.2% of the predicted hindgut SCFA production [50], the levels of SCFA in either serum or fecal samples were not able to reflect their SCFA-producing capacity and metabolic activity. An in vitro fermentation trial was performed to further test whether changes in fecal microbiota caused differences in SCFA-producing capacity and other metabolic activities. Results showed that gut microbiota in extruded dietary groups had different fermentation characteristics, e.g., lower SCFA levels, which agreed well with the lower abundance of commensal bacteria producing SCFA. This suggested the gut microbiota had important roles in regulating piglet feed intake when on different extrusion diets. However, why extruded ingredients induced such negative microbiota effects requires further investigation.

## 5. Conclusions

Substituting corn with extruded corn and extruded broken rice decreased piglet feed intake and growth performance and increased *Sarcina*, *Clostridium_sensu_strictio_1*, and *Terrisporobacter* pathogen levels. Based on the current findings, pig producers should be cautious when using extruded ingredients to formulate diets for weaned piglets. 

## Figures and Tables

**Table 1:** Diet composition and nutrient content (%).

Ingredients (%)	Treatments ^2^			
	CORN	ECORN	RICE	ERICE
Corn	62.17	31.59	31.63	31.65
Extruded corn		31.59		
Broken rice			31.63	
Extruded broken rice				31.65
Soybean	15.00	15.00	15.00	15.00
Extruded full-fat soybean	10.00	10.00	10.00	10.00
Fish meal (62.5% CP)	6.00	6.00	6.00	6.00
Soybean oil	2.00	1.00	1.00	1.00
Sucrose	2.00	2.00	2.00	2.00
L-Lysine (98%)	0.30	0.29	0.28	0.27
DL-Methionine (98.5%)	0.10	0.09	0.02	0.02
L-Threonine (98%)	0.04	0.03	0.03	0.02
L-Tryptophan (98%)	0.01	0.01	0.00	0.00
Choline chloride (50%)	0.15	0.15	0.15	0.15
Limestone	0.87	0.86	0.93	0.91
Dicalcium phosphate	0.65	0.65	0.60	0.60
Feed-grade sodium chloride	0.50	0.50	0.50	0.50
Vitamin and mineral premix ^1^	0.25	0.25	0.25	0.25
Total	100.00	100.00	100.00	100.00
Calculated nutrient content ^3^ (%)				
DE, Mcal/kg	3.522	3.521	3.523	3.519
CP (%)	19.02 (19.14)	19.13 (19.05)	19.19 (19.73)	19.20 (19.15)
Total lysine (%)	1.35 (1.34)	1.35 (1.31)	1.35 (1.33)	1.35 (1.32)
Total methionine (%)	0.39 (0.38)	0.39 (0.40)	0.39 (0.38)	0.39 (0.39)
Total Met + Cys (%)	0.73 (0.72)	0.73 (0.71)	0.74 (0.72)	0.74 (0.72)
Total tryptophan (%)	0.22 (0.20)	0.22 (0.21)	0.22 (0.20)	0.22 (0.21)
Total threonine (%)	0.79 (0.76)	0.79 (0.74)	0.79 (0.73)	0.79 (0.75)
Ca (%)	0.80	0.80	0.80	0.80
Total P (%)	0.65	0.66	0.60	0.62
ATTD P (%)	0.36	0.36	0.36	0.36

^1^ The premix provided the following vitamin and trace minerals per kilogram: vitamin A (transretiny lacetate), 15,000 IU; vitamin D_3_ (cholecalciferol), 2000 IU; vitamin E (all-rac-tocopherol-acetate), 40 IU; vitamin K (bisulfate menadione complex), 2.5 mg; riboflavin, 5 mg; pantothenic acid (d-Capantothenate), 15 mg; nicotinic acid, 30 mg; pyridoxine (pyridoxine HCl), 5 mg; thiamine (thiamine mononitrate), 2 mg; vitamin B_12_ (cyanocobalamin), 0.03 mg; d-biotin, 0.15 mg; folic acid, 1 mg; Se (Na_2_SeO_3_), 0.2 mg; I (KI), 1 mg; Cu (CuSO_4_5H_2_O), 160 mg; Fe (FeSO_4_7H_2_O), 225 mg; Mn (MnSO_4_H_2_O), 100 mg; and Zn (ZnSO_4_), 120 mg. ^2^ Treatments were corn as the main cereal type (CORN), or half of the corn replaced by extruded corn (ECORN), broken rice (RICE), and extruded broken rice (ERICE), respectively. ^3^ Values in the brackets are analyzed.

**Table 2:** Degrees of starch gelatinization in extruded/unextruded corn and broken rice.

	Corn	Extruded Corn	Broken Rice	Extruded Broken Rice
Degree of starch gelatinization (%)	12.74	75.48	14.76	93.46

**Table 3:** Effects of cereal types and extrusion processing on growth performance and diarrhea index of weaned piglets.

			Treatments ^2^			SEM		*p* Value	
		CORN	ECORN	RICE	ERICE		Cereal	Extrusion	Interaction
BW ^1^, kg	1 d	7.11	7.11	7.10	7.11	0.16	0.980	0.998	0.991
	14 d	10.27	9.70	10.41	9.82	0.24	0.610	0.187	0.995
	28 d	16.23	14.76	16.69	15.47	0.37	0.437	0.081	0.862
ADFI, g/day	1–14 d	401.2	376.8	415.2	370.2	16.4	0.791	0.580	0.844
	15–28 d	782.4	685.8	849.8	750.1	20.8	0.085	0.013	0.966
	1–28 d	591.8	536.3	632.5	561.0	17.0	0.376	0.054	0.718
ADG, g/day	1–14 d	221.2	183.1	230.8	196.0	13.8	0.723	0.256	0.983
	15–28 d	421.7	356.7	456.9	379.9	16.2	0.147	0.006	0.805
	1–28 d	323.4	262.9	346.3	285.4	11.7	0.35	0.015	0.818
F:G	1–14 d	1.81	1.97	1.8	1.89	0.1	0.853	0.101	0.762
	15–28 d	1.85	1.92	1.86	1.96	0.07	0.649	0.482	0.831
	1–28 d	1.83	1.95	1.83	1.94	0.08	0.773	0.419	0.805
Diarrhea index	1–14 d	1.17	0.89	1.02	0.93	0.12	0.794	0.275	0.863
	15–28 d	0.47	0.39	0.41	0.32	0.09	0.897	0.189	0.796
	1–28 d	0.82	1.95	1.83	1.94	0.06	0.847	0.216	0.807

^1^ ADFI, average daily feed intake; ADG, average daily growth; BW, body weight; FCR, feed conversion ratio; PWD, post-weaning diarrhea; SEM, pooled standard error of mean. ^2^ Treatments were corn as the main cereal type (CORN), or half of the corn replaced by extruded corn (ECORN), broken rice (RICE), and extruded broken rice (ERICE), respectively.

**Table 4:** Effects of cereal types and extrusion processing on the nutrient apparent digestibility and intake of digestible nutrients of weaned piglets.

		Treatments ^2^			SEM		*p* Value	
	CORN	ECORN	RICE	ERICE		Cereal	Extrusion	Interaction
ATTD of nutrients								
ATTD ^1^ of DM, %	85.07	84.57	86.96	86.63	0.42	0.021	0.611	0.921
ATTD of CP, %	78.79 ^a^	83.83 ^b^	82.61 ^b^	82.74 ^b^	0.68	0.269	0.042	0.049
ATTD of EE, %	74.56	71.16	77.28	72.45	1.05	0.336	0.055	0.731
ATTD of Ash, %	63.41	63.49	65.30	65.90	0.96	0.288	0.865	0.896
ATTD of GE, %	83.36	84.87	86.61	86.08	0.53	0.086	0.476	0.462
Intake of digestible nutrients								
DM, g/day	683.1	567.4	734.6	621.7	32.3	0.109	<0.001	0.965
CP, g/day	137.5	109.6	151.5	120.1	6.9	0.09	<0.001	0.803
Energy, Kcal/day	3110.6	2531.6	3485.8	2824.0	157.8	0.045	<0.001	0.795

^1^ ATTD, apparent total tract digestibility; DM, dry matter; CP, crude protein; EE, ether extract; Ash, crude ash; GE, gross energy; SEM, pooled standard error of mean. ^2^ Treatments were corn as the main cereal type (CORN), or half of the corn replaced by extruded corn (ECORN), broken rice (RICE), and extruded broken rice (ERICE), respectively. ^a,b^ Means in the same row with different letters differ significantly at *p* < 0.05. *n* = 8.

**Table 5:** Effects of cereal types and extrusion processing on levels of glucose, insulin, and ghrelin of weaned piglets.

	Cereal Type		Processing		Time				SEM	*p* Value						
	CORN	RICE	UE	E	0 h	1 h	2 h	4 h		C	E	T	C × E	C × T	E × T	C × E × T
Glucose, mmol/L	7.3	8.0	7.3	8.0	6.2	9.1	9.2	6.2	0.28	0.426	0.035	0.003	0.588	0.631	0.312	0.576
Insulin, mIU/mL	48.7	41.3	46.7	43.3	47.8	-	-	42.3	1.92	0.032	0.268	0.157	0.411	0.839	0.189	0.125
Ghrelin, mIU/mL	1726.4	1853.6	1759.8	1820.2	1621.4	-	-	1958.6	48.2	0.184	0.486	0.015	0.234	0.018	0.372	0.224

UE, unextrusion; E, extrusion; C, cereal type; T, time of collection; C × E, interaction between C and E; C×T, interaction of C and T; E × T, interaction of E and T; C × E × T, interaction of C, E, and T. Data are given as means and SEM, *n* = 8.

**Table 6:** Effects of cereal types and extrusion processing on the fecal concentrations of SCFA (mmol/L) of weaned piglets.

		Treatments ^1^			SEM		*p* Value	
	CORN	ECORN	RICE	ERICE		Cereal	Extrusion	Interaction
Acetate	40.04	42.88	48.06	42.39	1.99	0.370	0.734	0.311
Propionate	25.36	25.24	27.81	25.40	1.04	0.575	0.586	0.623
Butyrate	13.74	14.48	17.55	17.12	0.88	0.272	0.537	0.823

^1^ Treatments were corn as the main cereal type (CORN), or half of the corn replaced by extruded corn (ECORN), broken rice (RICE), and extruded broken rice (ERICE), respectively. SCFA, short-chain fatty acid. Data are given as means and SEM, *n* = 8.

**Table 7:** Effects of cereal types and extrusion processing on the plasma concentrations (μmol/L) of SCFA of weaned piglets ^1^.

	Cereal Source		Processing		Time		SEM	*p* Value						
	CORN	RICE	UE	E	0 h	4 h		C	E	T	C × E	C × T	E × T	C × E × T
Acetate	70.5	61.3	68.2	63.6	63.2	68.6	1.9	0.002	0.094	0.030	0.610	0.426	0.788	0.561
Propionate	59.6	60.6	62.2	57.9	62.5	57.7	2.5	0.701	0.318	0.252	0.819	0.038	0.094	0.219
Butyrate	10.9	8.3	10.2	9.0	11.2	8.0	0.3	<0.001	0.237	0.001	0.320	0.143	0.242	0.115
Total SCFAs	139.7	126.6	140.7	125.6	133.8	132.5	1.8	0.008	0.074	0.854	0.713	0.062	0.088	0.997

^1^ UE, unextrusion; E, extrusion; C, cereal type; T, time of collection; C × E, interaction between C and E; C × T, interaction between C and T; E × T, interaction between E and T; C × E × T, interaction of C, E, and T. SCFA, short-chain fatty acid. Data are given as means and SEM, *n* = 8.

**Table 8:** In vitro fermentation characteristics of the fecal microbiota.

			Treatments ^2^			SEM		*p* Value	
		CORN	ECORN	RICE	ERICE		Cereal	Extrusion	Interaction
In vitro fermentation characteristics ^1^	*V*_F_, mL/g	312.55 ^b^	310.35 ^b^	329.73 ^c^	284.93 ^a^	4.38	0.442	<0.001	0.001
	*K*, h^−1^	0.20	0.16	0.20	0.11	0.012	0.015	<0.001	0.055
	*F_RD0_*, h^−1^	0.10	0.08	0.10	0.05	0.006	0.011	<0.001	0.042
	*T*_1/2_, h	0.79	0.77	0.78	0.74	0.005	<0.001	<0.001	0.060
In vitro production of SCFAs, mmol/L	Acetate	29.29	24.44	28.31	22.65	0.89	0.375	0.002	0.791
	Propionate	8.10 ^a,b^	9.41 ^c^	8.91 ^b,c^	7.67 ^a^	0.38	0.254	0.933	0.004
	Butyrate	8.77 ^b^	9.41 ^c^	7.80 ^b^	6.44 ^a^	0.06	0.031	<0.001	0.006
	Isobutyrate	1.26 ^b^	1.11 ^b^	1.55 ^c^	0.76 ^a^	0.24	0.653	<0.001	<0.001
	Valerate	2.41 ^c^	1.65 ^b^	2.24 ^c^	0.92 ^a^	0.11	<0.001	<0.001	0.006
	Isovalerate	2.41 ^c^	2.07 ^b^	2.99 ^d^	1.55 ^a^	0.12	0.776	0.000	<0.001
	Total SCFA	52.24	46.80	53.00	39.98	1.48	0.208	0.001	0.118

^1^*V*_F_, the final asymptotic gas volume (mL/g feces); *FRD*_0_, initial fractional rate of degradation at t-value = 0 (h^−1^); *K*, fractional rate of gas production at particular time (h^−1^). *T*_1/2_, half-life to asymptote (h). ^2^ Treatments were corn as the main cereal type (CORN), or half of the corn replaced by extruded corn (ECORN), broken rice (RICE), and extruded broken rice (ERICE), respectively. ^a,b,c,d^ Means in the same row with different letters differ significantly at *p* < 0.05. Data are given as means and SEM, *n* = 5–8.

**Table 9:** The alpha diversity of fecal microbiota.

		Treatments ^1^			SEM		*p* Value	
	CORN	ECORN	RICE	ERICE		Cereal	Extrusion	Interaction
Coverage	0.998	0.998	0.998	0.998	0.00	0.604	0.412	0.902
Chao1	510.98	587.58	553.70	592.97	14.05	0.434	0.051	0.557
ACE	510.94	575.51	552.21	585.79	13.25	0.324	0.066	0.551
Shannon	3.14	3.49	3.57	3.45	0.11	0.387	0.608	0.310
Simpson	0.17	0.12	0.12	0.11	0.016	0.354	0.420	0.542

^1^ Treatments were corn as the main cereal type (CORN), or half of the corn replaced by extruded corn (ECORN), broken rice (RICE), and extruded broken rice (ERICE), respectively. Data are given as means and SEM, *n* = 8.

## Data Availability

The data in the present study are available on reasonable request from the corresponding author.
